# Annals of Quackery—III

**Published:** 1910-07-02

**Authors:** 


### ANNALS OF QUACKERY—III.

One of the most notorious of the quacks who flourished at the beginning of last century was "Dr." Eady. Of him it is said that "of all the impudent, audacious, and ignorant fellows that ever disgraced English credulity, this quack takes the lead." He was first an errand-boy, then went behind the counter of a haberdasher, and afterwards took a shop of his own, but finding trade bad he commenced business as a quack. When he first came to London he could not afford to pay for advertising his name in the papers, so regularly at nightfall he started from his lodgings and chalked with his own hand every public wall he came to until about one or two o'clock in the morning. He soon began to feel the benefit of these nightly excursions, and then commenced a regular advertisement campaign in the newspapers, in which it was set forth as follows: "Such is the effect of perseverance and industry in the science of medicine that great numbers, many of whom have lingered under the most painful and obstinate disorders, who are pronounced incurable by respectable practitioners, and several discharged from hospitals, have been restored to perfect health by the celebrated Dr. Eady," etc., etc. One cannot but be struck by the resemblance this bears to the advertisements of quack nostrums of to-day. In a subsequent issue of the *Medical Adviser* to that in which the attack was made appears the following announcement under the heading of "Notices to Correspondents"; "Eady, the quack, is positively deranged. His complaint is of a most variable and incurable character, sometimes assuming a religious melancholy, at others violent rage; and he frequently sits whole days in a comatose state. Considering this awful visitation, we really regret the severity of our attack upon him; but it was a duty we owed the public!"

### Goss and Company.

Of all the attacks which the *Medical Adviser* made, none was so bitter as that against the firm of "Goss and Company." This firm, or rather the man who adopted this title, was, "with his brother an understrapping scene-dauber with a strolling company." He relied upon the effect of his advertisements, which were got up in a most pompous and affected style calculated to impress the ignorant reader, as Goldsmith's talker on cosmogony imposed upon his Vicar of Wakefield, headed with a Latin quotation and filled with inflated bombast. One of these runs as follows: "*Carpe diem!—Be promptly wise.* When science began to explore the complicated machinery of man she was speedily entangled in a labyrinth of mysterious confusion, from which intellectual perseverance alone emancipated her. Ignorance and empiricism, however, still oppose their venomous hostility, and the prejudice of weak minds in some measure encourages their audacity. As truth is to falsehood, so may our public statements be compared with their unqualified pretensions, and to expose their fallacies we have not hesitated to avow ourselves as practitioners in a class of disease which endangers the mystic evolutions of nature; to be brief, we are members of the London College of Surgeons. The intricate and much-neglected disease," etc., and so on in this strain to some considerable length. Here and there between the phrases of this advertisement the editor of the *Medical Adviser* interpolates such remarks as "The only truth in your advertisement, Mr. Goss," which appears in brackets after the statement "the prejudice of weak minds encourages their audacity"; the following cryptic symbols, "a li*, a d— li*" are bracketed after the statement "we are members of the London College of Surgeons." It is not uninteresting to learn that "this mock company had the audacity last Saturday to indict Mr. Shackwell, the printer of the *Medical Adviser*, for an alleged libel therein contained." In a future issue, however, it appears that the "Grand Jury, men of good honest English sense, ignored the bill."

### The Whitworth Doctors.

It goes without saying that the quacks in those days were not a class peculiar to London only, and some interesting details are forthcoming concerning irregular practitioners in the provinces. There were at that time several living near Rochdale, in Lancashire, who were known by the name of Whitworth doctors. One proof of their superior knowledge was that a man in their village called Mr. Bradford was for a long time "plagued with live frogs in his belly," for the cure of which he had consulted three doctors, who told him it was impossible and that it was an imaginary evil; but the man was not satisfied by this explanation, for he "felt them in his inside." He immediately went to consult these Whitworth "doctors," who heard his symptoms very patiently and then told him they could cure him, to effect which they administered a strong emetic, and, wonderful to relate, "he pulled up three gert frogs, and has been hearty ever since he gat shut on 'em."

These Whitworth "doctors" did not take the comments of the *Medical Adviser* very kindly; in fact, they wrote to the editor and told him that the uncle of one of them "was sent for by his late Majesty to cure a polypus in the late Princess Amelia's nose, which he did, when all the London doctors had given her up as incurable." They also went on to say that "if the London quacks, as you call them, cannot find money to indict you, we will lay out a few thousands by way of a trial as the first. You talk of us curing a man that had live frogs in his belly, but we can cure you of having too few brains in your head!" Other provincial quacks referred to are Webster, of Liverpool, "who keeps a printing press at work instead of pestle and mortar, finding the composition of lies perhaps more lucrative than the trituration or compounding of drugs"; and Graham, a fellow living in an obscure den, yet figuring the walls of the town with large bills of "No Cure, No Pay."

The Footman Doctor.

There was, however, a woman in Kennington who outstripped all the quacks by advertising in large gilt letters upon a hoarding "All *incurable* diseases *cured.*" Reflecting upon the doings of "Doctor" Lynch, formerly a footman, who advertised to remove strictures of whatever nature merely by transmitting the affected believer a package of his medicine at the small charge of £5, paid in advance, the *Medical Adviser* is constrained to make the following very pointed observations: "Lynch has pocketed his five pounds, and the law cannot take it back—the law that can transport a wretch who obtains *five shillings* under any *other* false pretences but this, and that to save himself from starvation, while the culprit who thus pockets his five pounds is permitted to go unpunished, and thus accumulate still greater means of extending his infamous impositions throughout society. Why should this be? Are the members of Parliament amongst the credulous? Do they forget that quacks were only tolerated in early times because the science of medicine was scanty, and that the people were glad to have anything that professed a cure. Do they forget that we have a phalanx of physicians and surgeons who have arrived at the highest pitch of European medical knowledge? No, they do not; but the practice of public quackery has been since they can remember, and the flagrancy of its evils have never been exposed."

